# Activity profiles and hook-tool use of New Caledonian crows recorded by bird-borne video cameras

**DOI:** 10.1098/rsbl.2015.0777

**Published:** 2015-12

**Authors:** Jolyon Troscianko, Christian Rutz

**Affiliations:** 1 School of Biosciences, University of Birmingham, Birmingham B15 2TT, UK; 2 Department of Zoology, University of Oxford, Oxford OX1 3PS, UK

**Keywords:** animal-borne imaging, biologging, *Corvus moneduloides*, culture, extractive foraging, tool use

## Abstract

New Caledonian crows are renowned for their unusually sophisticated tool behaviour. Despite decades of fieldwork, however, very little is known about how they make and use their foraging tools in the wild, which is largely owing to the difficulties in observing these shy forest birds. To obtain first estimates of activity budgets, as well as close-up observations of tool-assisted foraging, we equipped 19 wild crows with self-developed miniature video cameras, yielding more than 10 h of analysable video footage for 10 subjects. While only four crows used tools during recording sessions, they did so extensively: across all 10 birds, we conservatively estimate that tool-related behaviour occurred in 3% of total observation time, and accounted for 19% of all foraging behaviour. Our video-loggers provided first footage of crows manufacturing, and using, one of their most complex tool types—hooked stick tools—under completely natural foraging conditions. We recorded manufacture from live branches of paperbark (*Melaleuca* sp.) and another tree species (thought to be *Acacia spirorbis*), and deployment of tools in a range of contexts, including on the forest floor. Taken together, our video recordings reveal an ‘expanded’ foraging niche for hooked stick tools, and highlight more generally how crows routinely switch between tool- and bill-assisted foraging.

## 1. Introduction

New Caledonian (NC) crows *Corvus moneduloides* use a range of tool types to extract embedded prey [1,2] and are the only non-human animals known to manufacture hooked tools in the wild [3]. While opportunistic observations have provided valuable glimpses of their complex tool behaviour [1,4,5], most quantitative information to date comes from field- and laboratory-based experiments, in which subjects are presented with artificial extraction tasks and a restricted choice of tools or tool materials (reviews: [2,6]). Given the difficulty of observing NC crows in the wild [7,8], innovative approaches are required to chart even basic aspects of their foraging ecology. For example, recent work investigated the energetics of ‘larva-fishing’ behaviour—where crows use (non-hooked) stick tools to extract giant wood-boring beetle larvae—by combining ‘camera-trap’ data from natural foraging sites [7] with stable-isotope-based estimates of diet composition [8].

Here, we build on our earlier work with miniature bird-borne video cameras to document the natural behaviour of wild, free-ranging NC crows [9]. Specifically, using our latest camera technology with much-improved functionality and recording capabilities [10], we aimed to collect a sufficient amount of video footage across subjects to produce first robust estimates of activity budgets. An assessment of the time spent foraging with and without tools is key to understanding the ecological significance, and possible evolutionary drivers, of tool behaviour [2,11]. By targeting a population for camera deployment where we had made two fleeting observations in 2007 of hooked stick tool manufacture and use, we tried to obtain the first field recordings of these behaviours. Surprisingly, little is known about how crows manufacture these particularly complex tools and which foraging substrates and prey types they target with them ([1,12]; all previous video footage was obtained at artificially baited feeding tables). Our results not only advance our knowledge of NC crows' foraging ecology, but they contribute more generally to documenting the adaptive value of animal tool behaviour—a topic of increasing research interest [2,8,11,13,14].

## 2. Material and methods

**Figure 1.**
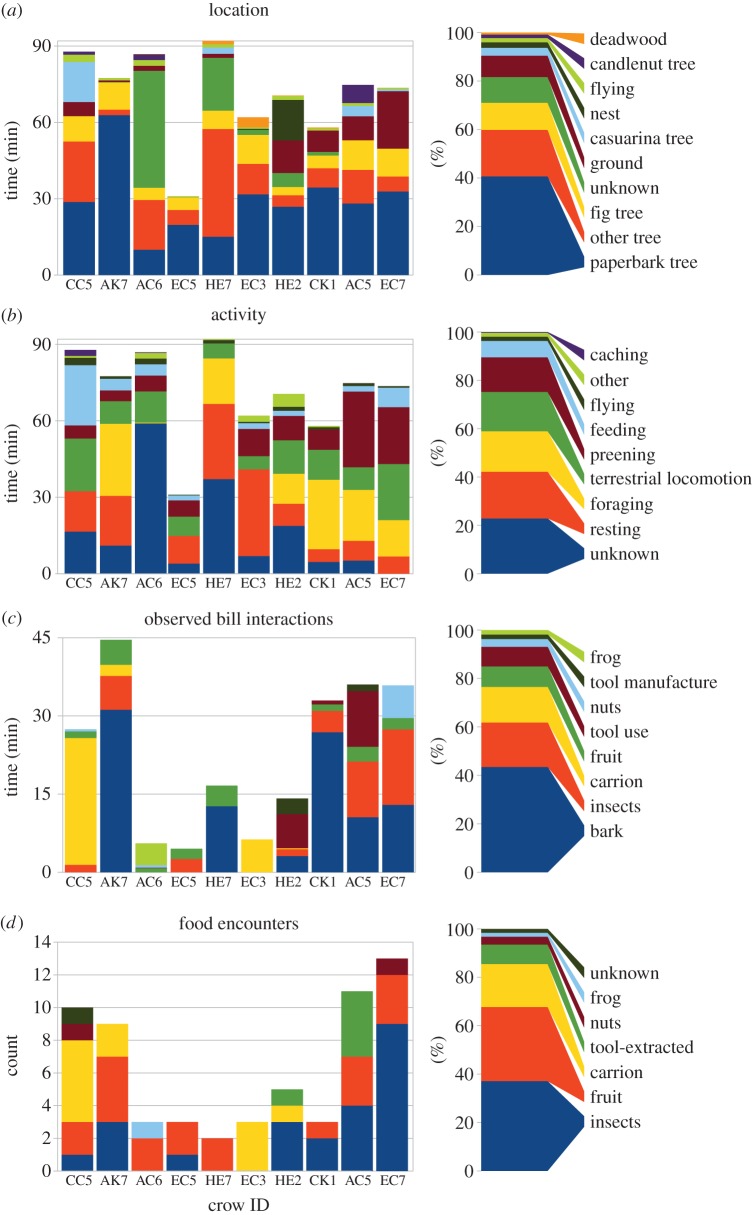
Activity profiles (*a*–*c*) and food encounters (*d*) of wild NC crows, as recorded by bird-mounted miniature video cameras. Bar charts show data for individual crows (left; identified by their ring codes) and percentage estimates for the whole sample (right).

We trapped 19 crows between 12 December 2009 and 18 January 2010 in our dry-forest study area in Gouaro-Déva, New Caledonia [7,8]. Loggers were customized (by choosing different battery and packaging options) to never exceed 5% of the birds' body mass (mean, 4.3%; range, 3.7–5.0%). We kept crows overnight in holding aviaries while building and programming their tags and released them the following day. Bait was removed from the study valley for every deployment to ensure that loggers filmed crows' natural foraging activities rather than visits to trap sites. Loggers were programmed to switch on in the morning, 1 day after release (to give birds time to habituate), and to record multiple bouts of footage following one of two different schedules (a 44-min morning session, with 2-min bouts every 28 min thereafter until battery depletion; a 30-min morning session and a 30-min late afternoon session on the first recording day, with 3-min bouts once every 17 min on the second day; for duty-cycling rationale, see [10]). As before (see schematic in [9]), cameras were attached to the two central tail-feathers to provide an ‘under-belly’ forward-directed view (see the electronic supplementary material, Movies S1 and S2). Rubber tubing held units firmly in place until UV degradation weakened the material and allowed safe detachment after an average of 7.3 days (range 3–15 days); an integrated miniature VHF radio-tag enabled the recovery of shed cameras. A detailed description of our video-logger technology and field procedures has been presented elsewhere [10]. All video footage was analysed at 1 s resolution using Media Player Classic (v. 6.4.9.1), a custom-written macro script (autohotkey v. 1.1.0), and a self-explanatory scoring scheme (figure 1).

## 3. Results

Ten out of 11 recovered video-loggers had successfully recorded data, yielding 714 min of crow-borne video footage (one unit had detached while recording). Figure 1 shows the location, behavioural activity, bill interactions and food encounters for all 10 subjects separately, together with corresponding summary plots (note that percentage estimates below refer to different samples of video footage, corresponding to figure 1*a–c*), and the electronic supplementary material, Movies S1 and S2 illustrate key behaviours.

**Figure 2.**
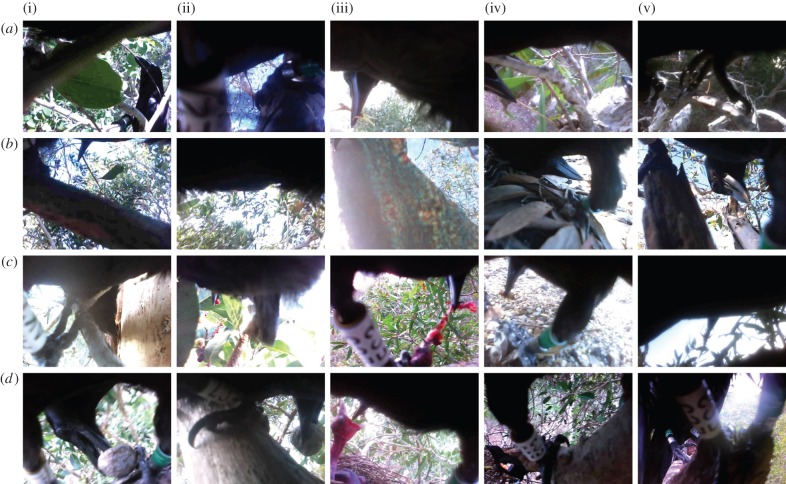
Still images from footage obtained with miniature video cameras attached to wild NC crows. For detailed image descriptions, see the electronic supplementary material, table S2. Panels *c*(ii) and *d*(iv) are reproduced with permission from [10].

Almost a quarter of all crow activity involved foraging (17%, defined as locomotion that to a human observer had no other apparent goal) or feeding (7%, defined as processing or eating food; figure 1*b*). Crows spent a significant amount of time in locally abundant paperbark trees *Melaleuca* spp. (41% of time; figure 1*a*), where they foraged by moving from branch to branch, inspecting holes and crevices, and peeling off sections of the soft, flaky bark (43% of bill interactions; figures 1*c* and 2*c*(i)). Prey obtained in this way included ants nesting within deadwood, wood-boring insect larvae (*ca* 1–2 cm long), small bright-green lepidoptera larvae (*ca* 0.5–1 cm long) and large adult insects (including cicadas). Crows were also observed foraging for, feeding on, or carrying around pieces of carrion, fruit and candlenuts (figure 2*c,d*), and one subject fed portions of a small frog to a begging juvenile.

Four crows were observed using tools in eight separate instances. Tool use—defined here as probing with a tool, bill-probing immediately following tool-probing in the same hole or crevice, and carrying tools—accounted for 8% of all observed bill activities, and tool manufacture for 2% (figure 1*c*); overall, 19% of all ‘foraging’ (figure 1*b*) involved tool-oriented behaviour. A number of instances were recorded where the crows' location and body movements were consistent with tool-probing but their bills were not visible. Ignoring these cases, it can be conservatively estimated that tool-oriented behaviour occurred in 3% of all recorded footage pooled across all 10 subjects, or 8% for the four tool-using subjects only; adjusting for ‘blackout’ periods (when the bird or vegetation obstructed camera view) only slightly increased these estimates.

The majority (96%) of tool use captured on video was in paperbark trees, accounting for about 21 min in six instances (by HE2, CK1 and AC5). All five (suspected) tool-derived prey items were obtained during paperbark foraging (owing to brief bouts of camera obstruction, the moment of extraction was not always witnessed), and included three to four medium-sized insect larvae and one large adult insect (possibly a cricket). In one instance, AC5 foraged with a hooked stick tool (see below) on the ground for a total of 53 s, probing through leaf litter and under a fallen branch. The only observation of tool-probing (with a non-hooked tool) in candlenut deadwood was a 6-second-long observation from AC6; feathers obstructed the camera's view before and after this brief glimpse, but the bird's posture and movements suggested that it foraged at this location for over 2 min.

Two instances of hooked stick tool manufacture were recorded (see the electronic supplementary material, Movie S2). Subject HE2 spent 179 s crafting a tool from a live paperbark twig. First, the bird snapped off the twig just above and below a branching node; it then stripped the bark and leaves from the longer, thinner branch and worked on the node to craft a hook. HE2 continued to probe into paperbark with this tool for the remainder of the programmed recording session (79 s). The second manufacture was recorded from AC5, which made a tool from a tree that looked like *Acacia spirorbis*. Construction took 59 s, from snapping off the branch, removing leaves and bark to fashioning the hooked end. The bird continued to probe with this tool for 690 s, moving through paperbark trees and probing in crevices and under bark, before probing in deadwood and leaf litter on the ground. This sequence also includes an instance where the bird dropped the tool onto the ground and promptly recovered it.

Video-loggers filmed unmarked crows a total of 21 times (counting a clutch of chicks as a single instance), and marked crows—identifiable from rings and wing-tags—a total of seven times; in one case, CC5 filmed an unmarked crow holding a tool (figure 2*a*(i)). Other sequences showed social interactions: for example, female EC3 repeatedly filmed its presumed partner, HE5 (figure 2*d*(iv),(v)), who: built a nest, which EC3 later inspected; appeared to be provisioning EC3 with food, possibly explaining EC3's comparatively high rest and low foraging rates (figure 1*b*); and was involved in side-by-side (allo-?) preening with EC3. HE2 was twice observed feeding newly hatched chicks on the nest with insects that it had collected on the ground (figure 2*d*(iii)).

## 4. Discussion

Although animal-borne video-loggers are still severely battery-limited and achieve only relatively modest data yields (in terms of footage recorded per unit deployed; [10]), they offer excellent research potential (in terms of biological insight gained per time and money invested; [9,15,16]). In our application, some 10 h of footage from 10 loggers have provided deeper insights into NC crow ecology than hundreds of hours of radio-tracking and opportunistic observations in the same study site. Compared to our earlier work with actively transmitting cameras [9], the use of duty-cycled solid-state loggers [10] has significantly improved our ability to collect uninterrupted recordings of behaviours of interest, which was key for achieving our principal study objective.

We present the first quantitative activity budgets for wild NC crows, and conservatively estimated that birds spent on average about 3% of their time handling and using tools (19% of foraging time), which is less than some of the values reported for woodpecker finches *Cactospiza pallida* [13]. Owing to the relatively short recording times per deployment, our video data cannot firmly establish whether individual crows differ in their reliance on tool-assisted foraging [8,13] (‘nil-returns’ may have simply been owing to chance)—this issue needs to be addressed with substantial observational datasets for a sample of subjects. Unlike in earlier years [7,8], freshly fallen, larva-infested candlenut logs were not available during the study period, so it was unsurprising that only one instance of tool use on an old candlenut log was filmed. Our observations of foraging behaviour in the absence of profitable larva-fishing opportunities are valuable for charting the broader ecological context of tool use in this species [2], as they highlighted, for example, crows' frequent switching between tool-assisted and bill-only foraging modes. The unambiguous documentation of hooked-stick-tool use in our long-term study site [7,8] suggests an expanded foraging niche for this tool type (all previous observations had been made in humid forest; [1,12]), and creates exciting opportunities to study the possible functional diversification of NC crows' stick tools (non-hooked versus hooked) in a habitat where they co-occur. Neither the manufacture of hooked stick tools from paperbark nor their deployment on the forest floor had been previously described.

In conclusion, our study makes two important contributions. Methodologically, it showcases how bird-borne video-loggers can be used to generate quantitative datasets for question-driven research—a clear indication of the coming-of-age of this cutting-edge technology [15,16]. In terms of biological insight, we have obtained the first quantitative estimates of the time NC crows spend foraging with, and without, tools, which is a crucial step towards identifying the ecological drivers of their remarkable tool behaviour (see §1 and [2,8]).

## Supplementary Material

Captions for Movies S1 and S2, and Tables S1 and S2

## Data accessibility

Data obtained through video scoring are shown in figure 1, and deployment information is summarized in the electronic supplementary material, table S1.
