# Supplementary material for: Activity profiles and hook-tool use of New Caledonian crows recorded by bird-borne video cameras
Source: Biol Lett. 2015 Dec;11(12):20150777. doi: 10.1098/rsbl.2015.0777 (PMC4707697; doi:10.1098/rsbl.2015.0777)
Supplement: Captions for Movies S1 and S2, and Tables S1 and S2 [file rsbl20150777supp1.pdf]

ELECTRONIC SUPPLEMENTARY MATERIAL

Activity profiles and hook-tool use of New Caledonian crows recorded by bird-borne video cameras

Jolyon Troscianko and Christian Rutz

- Captions for Movies S1 and S2
- Tables S1 and S2

MOVIE CAPTIONS

Movie S1. Sample clips of video footage recorded by miniature video cameras attached to wild New Caledonian crows, illustrating different habitats and (non-tool) foraging behaviours.

Movie S2. Sample clips of video footage recorded by miniature video cameras attached to wild New Caledonian crows, illustrating tool-oriented behaviour.

Table S1. Summary statistics for the deployment of miniature video cameras on 19 wild New Caledonian crows. Crows were uniquely marked with colour ID rings (3-digit alphanumerical codes, with the exception of RB73, which is a wing-tag code); sexed (F = female; M = male) using molecular techniques [Rutz *et al.* 2012, *Naturwissenschaften* 99, 313–320]; and aged (I = immature; A = adult) based on gape colouration [Rutz *et al.* 2010 *Science* 329, 1523–1526]. The estimate of video recordings refers to crow-borne footage (for EC5, the time when the logger was detached was not included), including blackout periods (i.e., when feathers or other objects obscured view, and behaviour could not be analysed; see Fig. 1).

date deployed	crow	sex	age	bird mass (g)	logger mass (g)	% of body mass	recovered?	date recovered	footage (min)	notes
12/12/2009	CC5	M	I	295	13.38	4.5	yes	22/12/09	88	good footage
12/12/2009	AK7	F	I	255	12.63	5.0	yes	22/12/09	77	good footage
12/12/2009	EK4	F	I	250	12.50	5.0	no	—	—	confirmed faulty VHF radio-tag
16/12/2009	HE8	M	I	360	13.29	3.7	no	—	—	cause of failure unknown
16/12/2009	AC6	M	A	315	13.09	4.2	yes	23/12/09	87	feather obscuring camera much of the time (see Fig. 1)
22/12/2009	HE5	M	A	350	13.39	3.8	no	—	—	confirmed faulty VHF radio-tag
24/12/2009	HC2	M	A	290	12.32	4.2	no	—	—	cause of failure unknown
24/12/2009	HE4	M	A	310	12.82	4.1	no	—	—	cause of failure unknown
24/12/2009	EC5	M	A	310	12.88	4.2	yes	30/12/09	31	camera detached before filming was complete (see Fig. 1)
27/12/2009	HE6	M	A	315	12.98	4.1	no	—	—	cause of failure unknown
28/12/2009	HC1	M	A	310	12.93	4.2	yes	not noted	—	no footage; packaging was damaged and camera head broken off
28/12/2009	HE7	M	I	330	13.07	4.0	yes	02/01/10	92	good footage, but camera tilted-up too much causing overexposure
31/12/2009	HC1	M	A	305	13.62	4.5	no	—	—	confirmed faulty VHF radio-tag
31/12/2009	EC3	F	A	255	12.51	4.9	yes	03/01/10	62	good footage, but camera head broken off
06/01/2010	HE2	M	A	340	13.09	3.9	yes	21/01/10	71	good footage
13/01/2010	EC2	F	A	265	12.56	4.7	no	—	—	did not detach before end of field season
14/01/2010	CK1	M	I	345	13.13	3.8	yes	21/01/10	58	good footage
15/01/2010	AC5	F	I	250	12.48	5.0	yes	21/01/10	75	good footage
18/01/2010	EC7	F	A	280	12.55	4.5	yes	21/01/10	74	good footage

Table S2. Description of still images from crow-borne video cameras, as shown in Fig. 2 in the main text. For details on crow subjects, and camera deployments, see Table S1.

image	crow	image content / crow behaviour
ai	AK7	camera-tagged bird films another (unmarked) crow holding a non-hooked stick tool
aii	HE2	hooked stick tool; note the neat hook at the end of the bill-held tool
aiii	HE2	manufacture of hooked stick tool from a paperbark branch; use of this raw material has not been previously described
aiv	HE2	insertion of hooked stick tool into substrate in search for arthropod prey
av	HE2	hooked stick tool
bi	AC5	manufacture of hooked stick tool (from *Acacia spirorbis*?)
bii	AC5	hooked stick tool
biii	AC5	hooked stick tool
biv	AC5	foraging with hooked stick tool on the forest floor; this behaviour has not been previously described
bv	AC5	foraging with hooked stick tool in standing deadwood
ci	AK7	bill-assisted foraging behind bark on a paperbark tree; note how the bird is pealing the bark away
cii	HE7	eating red berry (*Ficus* sp.?)
ciii	EC3	eating carrion
civ	HE2	capture of cicada on the ground
cv	CK1	capture of grasshopper in standing vegetation
di	EC7	eating cracked candlenut
dii	EC7	dropping candlenut at a nut-dropping site
diii	HE2	feeding of young chick in nest with arthropod prey
div	EC3	camera-tagged bird films another marked crow at a nest; the wing-tag combination identifies this bird as an adult male
dv	EC3	camera-tagged bird films another marked crow; the ring code (HE5) identifies this bird as an adult male
